# Enduring Challenges despite Progress in Preventing Mother-to-Child Transmission of Hepatitis B Virus in Angola

**DOI:** 10.3390/pathogens11020225

**Published:** 2022-02-08

**Authors:** Luis Baião Peliganga, Marco Aurélio Pereira Horta, Lia Laura Lewis-Ximenez

**Affiliations:** 1 Viral Hepatitis Laboratory, Oswaldo Cruz Institute, Oswaldo Cruz Foundation, Rio de Janeiro 21040-900, Brazil; peliluis@gmail.com; 2 Disease Control Department, National Directorate of Public Health, Ministry of Health, Luanda, Angola; 3 Internal Medicine Investigation Department, Faculdade de Medicina da Universidade Agostinho Neto, Luanda, Angola; 4 BSL-3 Facility, Oswaldo Cruz Institute, Oswaldo Cruz Foundation, Rio de Janeiro 21040-900, Brazil; marco.horta@fiocruz.br

**Keywords:** hepatitis B virus, elimination, mother-to child transmission, prophylaxis, newborn vaccination, pregnancy, vertical transmission, horizontal transmission, acute hepatitis B infection, Angola

## Abstract

Sub-Saharan Africa has one of the highest rates of hepatitis B virus (HBV) infection globally, with an incidence of 1.5 million and 0.8 million yearly deaths, which drives synergistic efforts towards its elimination. To assess the risk of mother-to-child transmission of HBV infection, a cross-sectional study was conducted on 1012 pregnant women in Angola to investigate HBV serological and molecular profiles. The prevalence of HBV was 8.7% (*n* = 88), with hepatitis B core IgM antibody (anti-HBc IgM) positivity identified in 12.8%, hepatitis B “e” antigen (HBeAg) positivity in 30%, and HBV DNA ≥ 200,000 IU/mL in 28.2%. Family tracking studied 44 children, of which 11 (25%) received at least two doses of the hepatitis B vaccine. HBV was detected in 10/44 (22.7%) children, with vaccination reported in one infected child. Further testing identified anti-HBc IgM positivity in 3/10 (30%), HBeAg positivity in 55%, and both seromarkers in 20%. The results revealed the importance of antenatal HBV screening, antiviral prophylaxis for mothers with high viral loads or HBeAg positivity, and timely first-dose hepatitis B vaccines in newborns. Anti-HBc IgM positivity among pregnant women and children highlights prophylactic measures worth considering, including antenatal hepatitis B vaccination and catch-up vaccination to young children.

## 1. Introduction

Sub-Saharan Africa (SSA) has one of the world’s highest prevalence of hepatitis B virus (HBV) infection. This infection, in 2019, accounted for 1.5 million new cases and 0.8 million deaths, which drives the need for synergistic efforts towards its prevention and treatment in key populations [1]. National programs in many countries in this region have implemented preventive measures to halt or decrease mother-to-child transmission (MTCT), with reports showing considerable progress throughout the years but still remain far from the target goals [2]. The shared characteristics of HBV and other transmission-related infections, such as syphilis and human immunodeficiency virus (HIV), allow for an integrated approach that facilitates the reduction of MTCT [3].

Since 2007, the World Health Organization (WHO) has recommended HIV and syphilis screening during antenatal consultations [4] and, more recently, HBV screening [2,5]. A growing number of countries have organized strategies for HBV testing along with syphilis and HIV, to increase the efficacy of antenatal care [1]. High-income countries successfully screen for these infections during pregnancy; however, limited access to antenatal care and diagnostic testing in low-income countries poses a challenge [6]. As the African region leads HIV testing and treatment, it wields the opportunity to extend this approach to HBV infection to reduce MTCT and the high burden of disease in the region [1]. Although many countries regularly perform syphilis screening together with HIV, HBV screening is yet to be incorporated as routine antenatal screening [6]. 

Angola elaborated an antenatal HBV screening policy that recommends testing during consultations [7] (Maria Furtado, Personal Communication, 10 December 2021), and antiviral prophylaxis with tenofovir disoproxil fumarate (TDF). The latter was included following the 2020 WHO recommendation for pregnant HBV-infected women with high concentrations of HBV DNA or positive surrogate seromarker for high viral replication, HBeAg to receive TDF to prevent MTCT [8]. It is important to note that this policy is being implemented nationwide. This study sought to identify the few remaining challenges in Angola and other SSA countries preventing the successful elimination of MTCT of HBV. 

## 2. Results

### 2.1. Population Profile

Between July and September 2007, 1012 pregnant women aged 13 to 46 years (average 24.5 ± 6.6), consented to participate in the study. The 20–29 age group accounted for 45.6% (462/1012) and 1.6% (17/1012) of participants did not know their age (Table 1). The gestational age varied from 4 to 42 weeks (average 25.6 ± 8.1), with 50.45% being in the third trimester of pregnancy. A total of 104 (10.2%) pregnant women were not registered for antenatal care and were in-patients that had been admitted for delivery or were at risk of a miscarriage. 

### 2.2. Screening for HBV, Syphilis, and HIV

All 1012 pregnant women were screened for the main serological marker for HBV infection, the hepatitis B surface antigen (HBsAg), of which 914/1012 (90%) were additionally tested for syphilis and HIV. The remaining 98 pregnant women were tested as follows: 65 for both syphilis and HBsAg, 2 for HIV and HBsAg, and 31 for HBsAg alone. Among the 1012 pregnant women tested for HBsAg, 88 (8.7%, 95% CI: 6.9–10.6) were positive, all of whom were confirmed using a different commercial rapid test, HBsAg Determine^®^/Abbott. Syphilis was detected in 69/979 (7.0%, 95% CI: 5.4–8.6); of these, 14 (20%) were retested with the rapid plasma reagin test with 100% concordance. HIV testing using HIV Vikia^®^ yielded 12 positive results; 10/12 (83%) were concordant with HIV1/2 Determine^®^ tests, and 9/12 (75%) were concordant with HIV 1/2 Unigold^®^. For this study, we considered at least two positive results; therefore, 10/916 patients (1.1%, 95% CI: 0.4–1.7) were considered to be HIV-infected. Co-infections with HBsAg and syphilis were identified in 6/979 (0.61%, 95% CI: 0.23–1.3), HBsAg and HIV in 1/916 (0.11%, 95% CI: 0.00–0.61), and syphilis and HIV in 3/914 (0.33%, 95% CI: 0.07–0.96).

### 2.3. Risk Factor Assessment

Seventeen participants lacked answers to the questionnaire, but were included for seroprevalence purposes. The average age was 23.5 ± 6.3 for the HBsAg-positive pregnant women and 24.7 ± 6.6 for all those that tested negative by all three tests (*n* = 769). Lack of antenatal care was higher among HIV-positive women 2/10 (20%) than among HBV-positive 9/88 (10.2%) or syphilis-positive 8/69 (11.6%) women, (χ^2^ = 0.86, N = 98, *p* = 0.35; and χ^2^ = 0.55, N = 79, *p* = 0.45, respectively); however, differences were not statistically significant. Demographic characteristics and risk factors for infected and non-infected pregnant women are outlined in Table 1. No significant risk factor was associated with HBV infection. The frequency of HBV infection was similar for age groups 13–19, 20–29, and over 40 years of age, yet, age group 30–39 carried the lowest frequency (Table 2).

### 2.4. Serological and Molecular Testing for HBV

**Figure 1:**
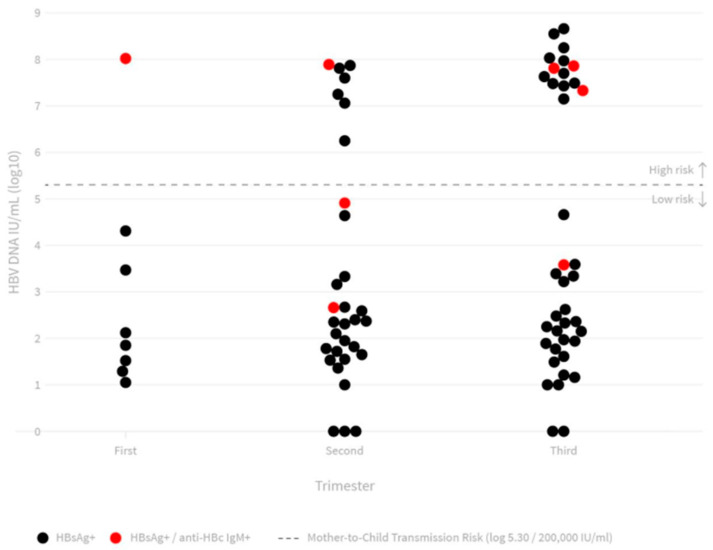
HBV DNA viral load distribution according to gestational age.

Anti-HBc IgM testing was conducted in 70/88 (80%) HBsAg-positive samples, HBeAg in 68/88 (77%) samples, and HBV DNA in 78/88 (89%) samples. Anti-HBc IgM and HBeAg were reactive in 9/70 (12.8%) in 21/68 (30.8%) samples, respectively. Of the 68 samples tested for both anti-HBc IgM and HBeAg, 5/68 (7.3%) were positive. HBV DNA was detected in 73/78 (93.5%) patients, with viral load varying from <10 IU/mL to 460,285,763 IU/mL. Twenty-two (28.2%) pregnant women had HBV DNA viral loads >200,000 IU/mL. There was no association between serological or molecular results obtained and age, gestational age, or coinfection. Despite this lack of association, the highest number of positive results for all serologies was seen in the age group 13–19 years (Table 2). Women in their third trimester had the highest prevalence of HBeAg and elevated HBV DNA viral load (Table 2 and Figure 1)**.** Syphilis co-infection was common among patients with positive serologies for anti-HBc IgM and HBeAg, and high HBV DNA viral load.

### 2.5. Offspring Tracking for HBV

Offspring were tracked in 14 (15.9%) of the 88 HBV-positive women. Four of these 14 mothers reported foetal or postpartum infant death of the child they were expecting at the time of recruitment in 2007. A total of 44 children from these 14 mothers were tested for HBsAg, with ages varying from 3 months to 21 years (average 6.4 ± 5.1), of which 38/44 (86.3%) were under 12 years of age. The gender distribution was 14 (31.8%) males and 30 (68.2%) females. Immunization status against hepatitis B was reported for 39/44 (88.6%) children, of which nine completed the three-dose scheme, two received two doses, and 28 had not been vaccinated. All 11 children who received the hepatitis B vaccine (three or two doses) were under two years of age. Ten (22.7%) of the 44 children tested positive for HBsAg and belonged to six HBsAg-positive mothers. HBsAg was detected in one child, who had completed the three-dose immunization scheme, whose mother was positive for both HBeAg and anti-HBc IgM, and carried a high concentration of HBV DNA (71,811,281 IU/mL). The remaining nine HBsAg-positive children had not been vaccinated against hepatitis B and were between 4–12 years of age. All HBV-positive children were further tested for anti-HBc IgM and HBeAg, except for one child that was not tested for HBeAg. Positive results for anti-HBc IgM were identified in 3/10 (30%) and HBeAg in 5/9 (55.5%). Dual positivity—anti-HBc IgM and HBeAg—was identified in 2/10 (20%) of the children (Table 3).

## 3. Discussion

### 3.1. Mother-to Infant (Vertical) Transmission

#### 3.1.1. Antenatal Care

In this study, 90% of pregnant women enrolled received antenatal care, which is higher than the 82% reported in Angola [9], and the 50% registered in other African countries [10]. This unusually high percentage observed in the study was because of the study recruitment location, the Hospital Geral do Bié’s antenatal care facility. Those lacking antenatal coverage were in-patients of the obstetric ward. The WHO recommends eight prenatal visits during pregnancy [11]; however, data from 20 countries in the sub-Saharan region, including Angola, on antenatal coverage of four or more antenatal visits, shows that no country in this region registered up to 80% coverage, and most countries had less than 50% [3,10]. Antenatal HBV screening is a key step to expanding this diagnostic investigation to the overall population and identifying women at risk for MTCT. However, lack of antenatal care results in these pregnant women not screened for HBV. Furthermore, the opportunity to administer timely preventive measures during pregnancy to avoid MTCT is lost [8,12]. Therefore, improving accessibility to antenatal care is crucial to achieving the goals recommended by the WHO to eliminate MTCT of HBV. 

#### 3.1.2. Screening for Triple Infections (HBV, Syphilis and HIV)

The SSA region is recognized as a leader in diagnostic testing for HIV [1], nonetheless the same cannot be said for syphilis and HBV [6]. Antenatal screening for hepatitis B has gradually increased in this region. Among the 27 WHO Member States from the African region that responded to the 2017 survey on Country Profile on Viral Hepatitis, 13 (48%) confirmed HBV testing in pregnant women [13]. 

In Angola, during the 2007 study period, antenatal screening for HIV and syphilis had already been implemented as a national policy, while HBV was recommended years later, in 2018. However, none of the study participants reported syphilis testing during antenatal consultations. This was most likely due to the irregularity in syphilis test supplies in the region at the time. Despite the delayed start in Angola, HBV screening is now well established in the capital and main cities, but is still insufficient throughout the country. Therefore, HBV infection among pregnant women is still underdiagnosed in Angola and other SSA countries [8,12].

The high prevalence (8.6%) of HBV detected in this study was similar to that detected (7.5%) by Sebastião et al. in pregnant women in Luanda, Angola, in 2018, despite the 11-year difference [14]. Other SSA countries have equally reported high HBV rates in pregnant women: Ghana, where prevalence ranged from 2.4–16.7%; Cameroon, 9.7%; and Nigeria, 10.5% [15,16,17]. Intermediate prevalence, has been reported in Ethiopia varying from 3 to 7.8% [18] as well as in Uganda and Tanzania with 2.9% and 3%, respectively [19,20]. In summary, intermediate to high and prevalence has been reported in the African region, which places the continent as one of the main regions with a high burden of HBV infection [19].

#### 3.1.3. Acute Hepatitis B Infection and Elevated Concentrations of HBV DNA

An important finding in this study was anti-HBc IgM positivity, a classical serological marker for acute HBV infection, identified among a few asymptomatic HBsAg-positive (9/70, 12.8%) pregnant women. The acute phase of HBV infection is typically characterized by an initial phase of elevated viremia [21], which poses a risk for intrauterine transmission [22,23]. In this study, among those anti-HBc IgM-positive who were tested for HBV DNA, 65% had HBV DNA concentrations > 7 log_10_ IU/mL. Since the anti-HBc IgM is detected 42 days after infection and can remain detectable up to six months, these pregnant women probably acquired infection during pregnancy or close to conception [24]. Four of the nine pregnant women with the acute condition at the time of the study were in their third trimester, and of these three with HBV DNA > 7 log_10_ IU/mL. HBV perinatal transmission during acute HBV infection occurs predominantly when the mother is infected during late pregnancy or during the first few months after delivery in 60% of cases [25]. As infant HBV immunization is less likely to prevent MTCT in these cases, a few countries have implemented HBV vaccination in pregnant women to protect them from infection during pregnancy. In Brazil, vaccination is recommended for all pregnant women who deny previous immunization or received incomplete coverage [26].

Elevated viral concentrations are not exclusive to the acute phase of HBV infection and are commonly found in chronic HBV infections, with or without the detection of the seromarker for high viral replication, HBeAg. This serological marker is a surrogate of elevated concentrations of HBV DNA, an important predictor for MTCT, especially when HBV DNA is above 200,000 IU/mL (>5.3 log_10_) IU/mL [8,27]. In this study, all HBeAg-negative pregnant women had viral concentrations below 200,000 IU/mL, and 18/20 (90%) HBeAg-positive women had a viral concentration greater than 200,000 IU/mL. Almost one-third (28.2%) of HBV-infected pregnant women had a viral load above 200,000 IU/mL. This high-risk profile for MTCT accounts for approximately 367,250 cases of perinatal HBV transmission per year in sub-Saharan Africa [28]. As an additional preventive measure, reducing HBV DNA levels below the transmission threshold through TDF prophylaxis at 28 weeks gestation has been a WHO recommendation since 2020 [8,29]. Keane et al. in their systematic review observed similar MTCT risks from HBeAg-positive mothers regardless of the child’s vaccination status, and at the time questioned the efficacy of timely birth doses [28]. As most of the mothers most likely had elevated levels of HBV DNA, intrauterine transmission may have occurred, and infant immunization was unable to prevent this form of transmission. 

#### 3.1.4. Timely Prophylaxis

Guidelines to prevent MTCT of HBV have established viral concentrations above 200,000 IU/mL or HBeAg-positivity as the standard to initiate prophylactic therapy [8,30,31,32] at the 28th week of gestation. The question arises as to what are the recommendations for health units that do not have access to these types of tests, as is quite common in the SSA region. Guinea-Bissau, located in western Africa, according to Dr. Adelino Gomes [33] (Personal Communication, 17 November 2021), ships HBV-positive samples to Portugal for further testing. If pregnant women detect HBV late in their pregnancy, the time frame for obtaining the test results may be cumbersome. Commercial rapid diagnostic tests for HBeAg detection have been considered as an option in a few low-income countries where laboratory settings are scarce; however, these tests have been shown to have low sensitivities to detect HBV DNA > 200,000 IU/mL [34,35]. Therefore, they are not suitable for defining prophylactic treatment eligibility during pregnancy. Brazil, like many African countries, has remote areas that lack these specific assays and cannot always rely on prompt test results from central public health laboratories. To overcome this problem, Brazil has recently included the policy of antiviral prophylactic therapy for all HBV-positive pregnant women without these specific tests, to be started between 28 and 32 weeks of gestation [36]. In this study, 10 pregnant women were diagnosed with HBV late in their gestation (>28 weeks) with HBV DNA concentrations >200,000 IU/mL, thus highlighting the importance of timely screening and initiation of prophylactic treatment to help eliminate MTCT.

#### 3.1.5. Neonatal Challenge

In addition to the one vaccinated HBV-infected child identified in this study, the authors LLL-X and LBP during clinical investigation in 2010 in Cuito, Bié, Angola, identified eight children HBsAg-positive who were under the age of two, whom had received partial or full hepatitis B vaccine schemes. The first dose of hepatitis B vaccine was administered at two months of age (unpublished data), since the HBV birth dose in Angola was introduced in 2014. The first dose of hepatitis B vaccine delivered within 24 h is the bases for prevention of vertical and horizontal transmission of HBV. Considering that this first dose should be administered at birth, the number of births that occur in health facilities is directly related to timely birth-dose coverage [3]. In the African region, an average of 42% of births occur at a health facility [37], and no more than 10% of the newborns receive their first dose of hepatitis B vaccine within 24 h [38]. During the 2015–2016 period, Angola reported that 46% of births occurred at health facilities, and the average timely HBV birth dose was delivered to 53.6% newborns, between 2016 and 2020 [9,39] (Alda Morais, Personal Communication, 1 August 2021). With the overall lack of health professionals to perform deliveries, midwives in this region have played an important role as skilled birth attendants and an integrated part of the group of maternal and newborn health professionals. These skilled birth attendants are most likely to assist in home deliveries and as such, are essential in the implementation of newborn immunizations in domestic settings. Indonesia has already implemented strategies where midwives store hepatitis B vaccines at home at room temperature for 30 days, and are able to deliver vaccines to newborns after birth in their homes [40]. This strategy should be considered in the African region to deliver timely first-dose hepatitis B vaccines to newborns born outside health facilities [41,42].

### 3.2. Intrafamiliar Horizontal Transmission

Early childhood-acquired HBV infections induce a state of immunotolerance of variable duration, with viral replication associated with a 15–25% risk of chronic liver disease-related mortality [18,32]. In this study, more than half of the infected children possessed the marker for high viral replication, HBeAg. These children, except for one, were not immunized against hepatitis B, as they had been born before this vaccine was introduced in Angola. 

Despite the availability of hepatitis B vaccines since 1981 and the recommendations by the WHO to implement immunization among infants since 1991, many countries with high prevalence have delayed the introduction of this vaccine as a nationwide policy. Angola, for instance, included hepatitis B vaccine in their national immunization program for infants in 2006 by using the pentavalent vaccine (diphtheria, pertussis, tetanus, hepatitis B, and *Haemophilus influenzae* type b), with reports [43] showing 83% complete three-dose coverage in their national infant immunization program. However, Angola, like many SSA countries that deferred the birth dose, did not include the mono-dose of hepatitis B vaccine until 2014, as recommended by the World Health Organization in highly endemic countries [44], and used combination vaccines to minimize costs and logistic expenses. Catch-up vaccination for unvaccinated children through national vaccine campaigns may decrease the number of susceptible young individuals, and should be considered in countries that register low vaccination coverage among infants.

Although strong efforts are being made to eliminate MTCT, domestic horizontal transmission of HBV is said to be one of the principal routes of HBV transmission in SSA [45,46]. Anti-HBc IgM, the seromarker of recent acute infection was identified in three of the infected children, aged 4, 5 and 7, suggesting intrafamiliar horizontal transmission. Additionally, the authors LLL-X and LBP during their clinical activity in 2010, were able to identify 45 children HBsAg positive, seven being positive for anti-HBc IgM and whose ages were four and 12 years (unpublished data). Furthermore, HBeAg was detected in 19 (90.4%) of the 21 children tested, suggesting high viral loads in these children.

HBV is stable and virulent in diverse environmental situations, and can maintain its infectivity outside the body from seven days to more than eight months [47]. This environmental stability together with high concentrations of virus are responsible for its high rate of infectivity. Although the highest concentrations of virus are detected in the blood, other body secretions such as saliva and wound exudates, etc., can also carry the virus, thus favouring environmental contamination [47]. Intrafamilial transmission occurs through contact between family members and a chronic carrier, frequently occurring in regions of high HBV endemicity, and may occur through interpersonal contact by sharing a toothbrush, sharp instruments, contact with exudates from dermatological lesions or contact with surfaces contaminated with HBsAg. It is estimated that 45% of people living with chronic carriers have a clear serology of past infection [48]. HBV has been found on the surface of toys, baby bottles, and skin lesion infiltrates in an environment where HBsAg-reactive individuals reside [49]. Intrafamilial transmission, especially from child to child, is also important in the natural history of the disease due to the high risk of chronicity in younger individuals. It is estimated that approximately 50% of infections in children are attributed to intrafamilial transmission in many endemic regions before vaccine introduction, where prevalence peaks in children aged 7–14 years [50]. 

### 3.3. Replicating Successful Programs to Eliminate MTCT

There are well-established government programs to control HIV infection, which is responsible for 37.7 million cases and 680,000 yearly deaths globally [1]. HBV, on the other hand, affects approximately 296 million individuals globally, corresponding to approximately 3.8% of the population, and accounts for approximately 820,000 deaths per year [2]. The HIV program´s vast experience in the logistics of testing and medication distribution has been shown to be effective, and the results are evident in many countries. For example, in Angola, hepatitis B vaccines and antiviral medication (TDF) are guaranteed through central government management. HBsAg testing kits are purchased by each municipality or province health department, thus putting the regularity of available screening tests at risk. Bearing in mind that most HBV surveillance and control programs are linked to HIV, the centralization of test and medication purchases and other supplies should follow the HIV model to ensure high coverage in health facilities.

### 3.4. Study Limitations

Considering that cross-sectional studies lack a temporal relationship between exposure and outcome, we were unable to confirm vertical or horizontal transmission among the offspring, as none were tested shortly after birth. Furthermore, laboratory testing was performed using only one sample, and thus, lacked sequential laboratory data for accurate diagnosis of acute HBV infection, since the detection of anti-HBc IgM is not exclusive to acute infection and is occasionally identified in acute exacerbations or “flares” of chronic infection. However, anti-HBc IgM values (cut-off ratios or titres) could help distinguish these different phases, as high values are associated with the acute phase of infection [51]. We identified two pregnant women whose anti-HBc IgM values were higher than the cut-off index value for acute HBV infection for the commercial assay used. Nonetheless, this does not necessarily classify all other results as acute exacerbations of chronic infection, since lower values can be found during the early anti-HBc IgM seroconversion period or during the late phase when the values tend to drop.

## 4. Materials and Method

### 4.1. Study Population

The Bié province, located in the central region of Angola, was one of the regions most affected by the civil war, and was partially destroyed and isolated between 1992 and 2002. Their central hospital, Hospital Geral do Bié, is the reference for over 2.8 million inhabitants. This cross-sectional study enrolled pregnant women from the Prenatal and Obstetrics Clinic of the Hospital Geral do Bié, Kuito, Angola. Before recruitment, explanatory lectures on hepatitis B, syphilis, and HIV were given in their local dialects, and informed consent was obtained. A standardized questionnaire was used to query demographic information and risk factors for parenteral (transfusion, surgery, use of unsterile needles in cultural rituals, and sharing of sharp objects) and sexual (number of sexual partners, condom use, previous history of sexually transmitted infections, and use of intravaginal astringent substances) exposure. Pregnant HBsAg-positive women were contacted two years later (2009) for follow-up of their children, and additional recruitment was carried out for children who tested HBsAg-positive for further blood draws to investigate hepatitis B serological markers.

### 4.2. Blood Specimens

#### 4.2.1. Immediate Testing and Referral

Blood samples were collected for storage and immediate testing, and a team physician provided the results on the same day. HBsAg was determined by a commercial rapid test HBsAg Vikia^®^ (bioMerieux/SA, bioMerieux^®^SA, Marcy l′Étoile, France), and all positive samples were retested using a second commercial rapid test (HBsAg Determine^®^/Abbott Japan Co.,Tokyo, Japan). All women who tested positive for HBsAg were referred to a local physician for further evaluation and testing, and newborn vaccination was recommended. Positivity to syphilis was determined by the rapid Syphilis Strip Test BioEasy^®^ and, when available, further confirmed by rapid plasma reagin (RPR) testing. Pregnant women with syphilis were treated with intramuscular benzathine penicillin. The rapid anti-HIV Vikia^®^ test (bioMerieux/SA, France) was used to determine anti-HIV positivity. All positive results were retested using two other commercial rapid tests, HIV1/2 Determine^®^ (Abbott Japan Co.,Ltda.,Tokyo, Japan) and Uni-Gold™HIV (Trinity Biotech Plc., Dublin, Ireland) and identified as positive if reactive in at least two tests. All women who tested positive for HIV were referred to local HIV counselling services for evaluation and treatment.

#### 4.2.2. Serological and Molecular Testing for HBV

Samples collected in 2007 were centrifuged, serum aliquoted into two cryotubes, and stored in a freezer at −30 °C at the Hospital Geral do Bié until they were sent to Brazil. The samples were shipped on dry ice to the Viral Hepatitis Laboratory/Oswaldo Cruz Institute/Oswaldo Cruz Foundation/Brazil and immediately stored at −80 °C until thawed for serological or molecular tests for HBV. HBsAg-positive samples were subjected to HBeAg and anti-HBc IgM testing through a commercial enzyme immunoassay (AxSYM; Abbott Diagnostics, Chicago, IL, USA) and the concentrations of HBV DNA were determined using a commercial quantitative PCR assay (Abbott RealTime HBV^®^ Abbott Molecular Inc., Des Plaines, IL, USA). As specimen volume could be insufficient to perform all three tests, priority was given to HBV DNA testing, followed by anti-HBc IgM and then HBeAg.

An identical procedure was carried out on blood drawn from the HBV-infected children in 2009, except that testing was limited to serological investigation (anti-HBc IgM and HBeAg).

### 4.3. Statistical Analysis

The basic characteristics of the dataset were summarized using descriptive statistics for variables age categories, gestational age and co-infection, and prevalence for hepatitis B serological (HBsAg, Anti-HBc IgM and HBeAg) and molecular markers (HBV DNA). The χ^2^ test was used to determine the relationship between variables and HBV positivity. Analyses were performed using RStudio 3.0.1 software (https://www.rstudio.com/products/rstudio/download/ accessed on 13 November 2021).

## 5. Conclusions

Despite the high seroprevalence for HBV identified in pregnant women; gradual improvements have been acknowledged in preventing MTCT in Angola. A few important limitations remain that may hinder the elimination of MTCT of HBV unless they are addressed accordingly: (1) the accessibility to antenatal care and HBsAg testing, (2) timely birth doses in newborns born outside health facilities, (3) delivery of hepatitis B vaccines to pregnant women, and (4) catch-up vaccination for young children.

## Figures and Tables

**Table 1:** Demographic characteristics and frequency of risk factors in pregnant women at the Prenatal and Obstetric Clinic/Hospital Geral do Bié, Kuito, Bié, Angola.

Characteristics	N	(%)	HBsAg Prevalence	OR	95% CI	*p* Value
Total	1012	(100)	8.7	-	-	-
Age category (years)						
13–19	273	(27.0)	9.5	1.01	0.35–2.86	0.981
20–29	462	(45.6)	9.3	1.05	0.12–8.55	0.962
30–39	250	(24.7)	6.8	0.72	0.08–6.04	0.770
≥40	11	(1.0)	9.1	1.0-		
Not known	16	(1.5)	6.2	0.66	0.08–11.93	0.783
Gestational age (weeks)						
1–12	84	(8.3)	10.7	1.32	0.61–2.82	0.471
13–24	412	(41.1)	8.9	1.04	0.82–1.31	0.722
25–42	505	(49.9)	8.3	1.0-		
Unknown	11	(1.0)	0.0	-	-	-
Risk Factors						
Antenatal care						
Yes	908	(89.7)	8.7	1.0-		
No	104	(10.3)	8.6	0.99	0.48–2.04	0.987
Alcohol intake						
Drinker	113	(11.4)	8.8	1.01	0.50–2.06	0.963
Non-drinker	883	(88.6)	8.7	1.0-		
Blood transfusion						
Yes	29	(2.9)	13.7	1.69	0.57–4.97	0.955
No	972	(97.1)	8.6	1.0-		
Surgery						
Yes	55	(5.5)	5.4	0.58	0.17–1.90	0.373
No	945	(94.5)	9.9	1.0-		
Tattoo						
Yes	30	(3.0)	16.6	2.14	0.79-5.74	0.130
No	972	(97.0)	8.5	1.0-		
Body piercing						
Yes	883	(88.1)	9.0	1.61	0.72–3.58	0.238
No	119	(11.9)	5.8	1.0-		
Scarification						
Yes	183	(18.3)	8.7	0.99	0.56–1.75	0.983
No	819	(81.7)	8.7	1.0-		
Female genital circumcision						
Yes	11	(1.1)	9.1	1.03	0.13–8.21	0.971
No	991	(99.9)	8.7	1.0-		
Razor sharing						
Yes	75	(7.5)	9.3	1.07	0.47–2.41	0.861
No	927	(92.5)	8.7	1.0-		
Sharing of nail grooming appliances						
Yes	458	(45.9)	8.0	0.84	0.54–1.31	0.449
No	540	54.1)	9.4	1.0-		
Sharing of toothbrush						
Yes	62	(6.2)	9.6	1.12	0.46–2.68	0.797
No	940	(93.8)	8.7	1.0-		
Use of intravaginal astringent substances						
Yes	500	(50.0)	8.0	0.81	0.52–1.27	0.372
No	500	(50.0)	9.6	1.0-		
Anal sex						
Yes	191	(19.1)	7.3	0.78	0.43–1.42	0.429
No	810	(80.9)	9.1	1.0-		
Oral sex						
Yes	147	(14.7)	8.1	0.90	0.48–1.71	0.771
No	854	(85.3)	8.8	1.0-		
More than one sexual partner in the past 6 months						
Yes	14	(1.4)	14.2	1.74	0.38–7.92	0.470
No	987	(98.6)	8.7	1.0-		
Polygamous sexual partner						
Yes	413	(54.0)	8.9	1.03	0.62–1.70	0.893
No	357	(46.0)	12.6	1.0-		
No or infrequent use of condoms						
Yes	977	(97.7)	8.9	2.15	0.28–16.14	0.457
No	23	(2.3)	4.35	1.0-		
Past history of STD						
Yes	99	(9.9)	12.1	1.49	0.78–2.85	0.223
No	900	(90.1)	8.4			
Syphilis						
Yes	69	(7.1)	8.7	1.01	0.42–2.42	0.972
No	910	(92.9)	8.5	1.0-		
HIV						
Yes	10	(1.1)	10.0	1.16	0.14 -9.29	0.887
No	906	(98.9)	8.7	1.0-		

N, number of pregnant women tested; HBsAg, hepatitis B surface antigen; STI, sexually transmitted infections.

**Table 2:** Hepatitis B serological and molecular markers.

Characteristics	HBsAg			Anti-HBc IgM			HBeAg			HBV DNA				
	N	Positive	(%)	N	Positive	(%)	N	Positive	(%)	Median	Log_10_ IU/mL (Average ± sd)	N	>200,000 IU/mL	(%)
Age category (years)														
13–19	273	26	(9.5)	17	3	(17.6)	17	7	(41.2)	2479	4.0 ± 2.9	23	8	(34.8)
2–29	462	43	(9.3)	36	3	(8.3)	36	10	(27.8)	228	3.4 ± 2.4	39	9	(23.1)
30–39	250	17	(6.8)	15	2	(13.3)	13	3	(23.1)	242	3.5 ± 2.9	14	4	(28.6)
≥40	11	1	(9.1)	1	0	(0)	1	0	(0)	36	1.5 *	1	0	(0)
Gestational age														
1st trimester	84	9	(10.7)	6	1	(16.7)	6	1	(16.7)	102	2.9 ± 2.3	8	1	(12.5)
2nd trimester	412	37	(9.0)	32	4	(12.5)	30	6	(20.0)	235	3.2 ± 2.5	31	7	(22.6)
3rd trimester	505	42	(8.3)	32	4	(12.5)	32	14	(43.7)	1666	4.1 ± 2.9	39	14	(35.9)
Coinfection														
Syphilis positive	69	6	(8.7)	5	1	(20.0)	5	3	(60.0)	3.59 × 10^7^	5.2 ± 3.2	6	3	(50.0)
negative	910	78	(8.6)	62	7	(11.3)	60	18	(30.0)	235	3.5 ± 2.6	71	19	(26.8)
HIV positive	10	1	(8.7)	0	-	-	0	-	-	133	2.1 *	1	0	(0)
negative	906	79	(10.0)	66	8	(12.1)	64	20	(31.2)	235	3.5 ± 2.7	73	20	(27.4)

HBsAg, hepatitis B surface antigen; anti-HBc, hepatitis B core antibody; IgM immunoglobulin class M; HBeAg: hepatitis B “e” antigen; N, number of pregnant women tested; sd, standard deviation; IU/mL, international units per millilitre; * only 1 registered case.

**Table 3:** Laboratory and vaccine profile of the HBsAg-positive mothers and their offspring with at least one HBsAg-positive child.

FAMILY	Profile	Age	Gender	HBsAg	HBeAg	Anti-HBc IgM	HBV DNA (10 log)	Vacine HBV
1	Mother	27	F	Positive	Positive	Neg	1.21	No
	Child 1	9	F	Neg	-	-	-	No
	Child 2	7	F	Positive	Positive	Neg	-	No
	Child 3	5	F	Positive	Neg	Neg	-	No
	Child 4	1	M	Neg	Neg	Neg	-	Yes
2	Mother	34	F	Positive	Neg	Neg	-	No
	Child1	16	F	Neg	-	-	-	No
	Child 2	9	F	Positive	Positive	Neg	-	No
	Child 3	4	M	Neg	-	-	-	No
	Child 4	1	F	Neg	-	-	-	Yes
6	Mother *	42	F	Positive	Neg	Neg	1.55	No
	Child 1	21	F	Neg	-	-	-	No
	Child 2	20	F	Neg	-	-	-	No
	Child 3	14	F	Neg	-	-	-	No
	Child 4	13	F	Neg	-	-	-	No
	Child 5	10	F	Positive	Neg	Neg	-	No
	Child 6	8	F	Neg	-	-	-	No
7	Mother	27	F	Neg	Neg	Neg	3.16	No
	Child 1	10	M	Neg	-	-	-	No
	Child 2	8	F	Neg	-	-	-	No
	Child 3	7	F	Neg	-	-	-	No
	Child 4	4	F	Neg	-	-	-	No
	Child 5	1	F	Neg	-	-	-	Yes
8	Mother	17	F	Neg	Neg	Neg	2.10	No
	Child 1	1	F	Neg	-	-	-	Yes
9	Mother *	28	F	Positive	Neg	Neg	-	No
	Child 1	10	M	Neg	-	-	-	No
	Child 2	6	F	Neg	-	-	-	No
	Child 3	5	M	Neg	-	-	-	No
	Child 4	3	F	Neg	-	-	-	No
	Child 5	3m	F	Neg	-	-	-	incomplete
10	Mother	21	F	Positive	Positive	Positive	7.86	No
	Child 1	1	M	Positive	Positive	Neg	-	Yes
11	Mother *	20	F	Positive	Neg	Neg	2.62	No
	Child 1	5	M	Positive	Positive	Neg	-	No
	Child 2	4	F	Positive	Positive	Positive	-	No
	Child 3	8m	F	Neg	-	-	-	Yes
15	Mother	29	F	Positive	Neg	Neg	1.0	No
	Child 1	1	F	Neg	-	-	-	Yes
16	Mother	17	F	Positive	Neg	Neg	-	No
	Child 1	1	M	Neg	-	-	-	Yes
17	Mother *	23	F	Positive	Neg	Neg	1.29	No
	Child 1	5	F	Neg	-	-	-	No
	Child 2	3	M	Neg	-	-	-	unknown
28	Mother	28	F	Positive	Neg	Neg	-	No
	Child 1	12	M	Positive	Neg	Neg	-	No
	Child 2	7	F	Positive	Neg	Positive	-	No
	Child 3	5	F	Positive	Positive	Positive	-	No
	Child 4	2	F	Neg	-	-	-	unknown
31	Mother	37	F	Positive	Neg	Neg	-	No
	Child 1	14	F	Neg	-	-	-	No
	Child2	10	F	Neg	-	-	-	No
	Child3	8	M	Neg	-	-	-	No
	Child 4	4	F	Neg	-	-	-	No
	Child 5	2	M	Neg	-	-	-	Yes
107	Mother	23	F	Positive	Neg	Neg	3.33	No
	Child 1	2	M	Neg	-	-	-	unknown
	Child 2	3m	F	Neg	-	-	-	Yes

*, miscarriage or child deceased shortly after birth; HBsAg, hepatitis B surface antigen; anti-HBc, hepatitis B core antibody; IgM, immunoglobulin class M; HBeAg: hepatitis B “e” antigen; m, months; F, female; M, male; Neg, negative; “-” not tested.

## Data Availability

Data reported in this study are available upon request.
